# Supplementary material for: Functional diversity of sharks and rays is highly vulnerable and supported by unique species and locations worldwide
Source: Nat Commun. 2023 Nov 24;14:7691. doi: 10.1038/s41467-023-43212-3 (PMC10673927; doi:10.1038/s41467-023-43212-3)
Supplement: Supplementary file 1 — Supplementary Information [file 41467_2023_43212_MOESM1_ESM.pdf]

**Supplementary Information for**

# Elasmobranch functional diversity is highly vulnerable and supported by unique species and locations worldwide

Catalina Pimiento, Camille Albouy, Daniele Silvestro, Théophile L. Mouton, Laure Velez, David Mouillot, Aaron B. Judah, John N. Griffin*, Fabien Leprieur*
Email: catalina.pimientohernandez@pim.uzh.ch
*These authors contributed equally

**Table of contents**

Supplementary Methods 2
- Species data. 2
- Trait data. 2

Supplementary Figures 6
- Figure S1. Distribution of trait categories per Order. 6
- Figure S2. Functional space occupied by the two elasmobranch superorders. 7
- Figure S3. Functional space occupied by the different IUCN Red List categories. 8
- Figure S4. Elasmobranch extinction risk. 9
- Figure S5. Trait correlates of extinction risk. 10
- Figure S6. Top 20 species 11
- Figure S7. Spearman correlation between metrics. 12
- Figure S8. Three-dimensional elasmobranch functional space. 12
- Figure S9. Relationships between biodiversity indices. 13
- Figure S10. Other facets of elasmobranch biodiversity and residuals 14
- Figure S11. Distribution of species-based metrics. 15
- Figure S12. Hotspots of elasmobranch diversity and their overlap 16
- Figure S13. Draftsman plot for all the biodiversity metrics. 17
- Figure S14. Cumulative Fishing Pressures (2012-2020). 17
- Figure S15. Schematic representation of our assessment of MPAs. 18
- Figure S16. Elasmobranch biodiversity represented in the world's MPAs. 19
- Figure S17. Elasmobranch biodiversity coverage by the world's MPAs. 20
- Figure S18. Percentage of biodiversity hotspots protected by MPAs. 21
- Figure S19. Correlation between imputed trait values across 10 iterations. 22

Supplementary Tables 23
- Table S1. Quality of the functional spaces 23
- Table S2. Relationships between each PCoA axis and the original trait values. 23
- Table S3. Functional diversity and species richness by superorders and orders. 24
- Table S4. Per-species FSp, FUn, FUSE, ED2 and EDGE2 across orders 24
- Table S5. Elasmobranch functional and species richness by IUCN status. 25
- Table S6. Binomial GLM output for the effect of traits on extinction probabilities 25

References cited in this Supplementary Information file 26

**Supplementary Methods**

**Species data.**
The following species were excluded from our analyses:

- Species absent from the phylogenetic tree from[1]: *Aetobatus ocellatus, Aetomylaeus milvus, Apristurus breviventralis, A. garricki, A. nakayai, Miroscyllium sheikoi, Breviraja mouldi, Carcharhinus humani, Centrophorus uyato, Hemiscyllium halmahera, Himantura bleekeri, Pristiophorus lanae, Rajella paucispinosa and Tetronarce cowleyi.*
- Sixty-seven species without spatial information (see main text).
- Sixty species from which trait information was not available.
- *Myliobatis aquila* was excluded from our analyses because it only occurs in cells with less than four species, which was a prerequisite to perform functional diversity analyses given the number of dimensions of the functional space (see main text).

**Trait data.**
Trait data was downloaded from FishBase (www.fishbase.org/; last accessed January 2019) using the R package "rfishbase"[2] and complemented with data gathered from the IUCN Red List of Threatened Species (www.iucnredlist.org/; last accessed October 2021). After the data download, the trait information was carefully checked against primary literature and updated (Dataset S1; Text S1).

The following traits were collected:

*Maximum body size*: In sharks, body size is expressed as total length (TL), estimated as the distance from the tip of the snout to the posterior end of the dorsal caudal lobe. In batoids (except rhinopristids, some skates, and some rays from the family Urolophidae; see below), the width of the disc (WD) is estimated as the distance between the wing tips (accordingly, the tail and rostrum lengths of batoids are not considered). Maximum body size was expressed as the largest TL or WD values recorded for each species. This trait was numerical and log-transformed.

*Habitat*: We categorized the habitat where organisms live based on the zone where they occur as adults. Trait designations were as follows:

- Coastal: Living on or around continental and insular shelves (category = "shelf" in Dataset S1). These species are inshore and coastal and are commonly found between depths of 0-200m.
- Oceanic: Living beyond shelves and slopes, and on or around continental and insular slopes (category = "slope" and "offshore" in Dataset S1). These species are commonly found in depths greater than 200m. Species from offshore seamounts, submarine canyons and ridges are also considered oceanic as well as those existing on the abyssal plain.

These data were gathered from databases and the primary literature (see Dataset S1 and Text S1). For the analyses, this trait was re-coded to be binary and fuzzy, as a single species could have multiple values (e.g., *Carcharodon carcharias*: coastal =1; oceanic = 1; see Dataset S2).

*Vertical position* Vertical position was assigned based on the most frequent part of water column where they feed. Trait designations are as follows:

- Benthic: Feeding on or near the seabed, may also contain substrate preferring demersal species.
- Pelagic: Feeding mainly along the water column.
- Benthopelagic: Feeding on both, the seabed and the water column.

These data were gathered from databases, guides and the primary literature (see Dataset S1 and Text S1). For the analyses, this trait recoded to be binary and fuzzy, as a single species could have multiple values (e.g., *Carcharias taurus:* benthic = 1, pelagic = 1).

*Terrestriality:* This trait differentiates between species which are exclusively marine or can enter brackish or freshwater [3]. The trait designations are as follows:

- Marine: fully marine species that do not interact with the terrestrial realm.
- Brackish: Species that can enter estuaries, or other brackish habitats.
- Freshwater: Species that enter rivers or that migrate between freshwater and the sea.

All fully freshwater species were excluded (e.g., *Potamotrygon falkneri, Potamotrygon schroederi* and *Potamotrygon tigrina*). This trait was ordinal (marine > brackish > freshwater).

*Thermoregulation:* Thermoregulatory strategies in elasmobranchs can take two forms:

- Mesothermic: Animals that can control the temperature of some of their most important organs, also called regional endothermy. The trait "mesothermic" is restricted to certain taxa and was assigned to all lamnid sharks (family Lamnidae) and a species of thresher shark (*Alopias vulpinus*) [4, 5, 6, 7, 8, 9, 10]
- Ectothermic: Animals incapable of self-regulating their body temperatures. This is the most common physiological strategy among fishes. The trait value "ectothermic" was assigned to all remaining sharks and rays, including *Mobula tarapacana* and *Manta birostris* [11]. The last two species possess an anatomical characteristic (i.e., *retia mirabilia*) that indicates the presence of a heat-exchange system [12, 13] and the first species is known to have the ability to dive into waters of < 4°C [14]. These characteristics have suggested the possibility of a mesothermic capability for these two species of rays. However, we have considered them to be ectothermic because: 1) it has been established that there is no reliable temperature measures to confirm that the *retia* in these species actually maintains a higher internal temperature relative to the sea temperature [11]; 2) more extreme dives (hence, tolerance to colder waters) have been reported in ectothermic sharks such as the whale shark shark [14] and have been explained by behavioural thermoregulation [15] and their large body size [16] rather than by regional endothermy; and 3) it has been empirically demonstrated that the ecological benefit of mesothermy in fishes, an energetically demanding feature, is enhanced predation (via elevated cruising speeds) [17], which contrasts with the filter feeding *M. tarapacana* and *M. birostris* (both tropical species).

This trait was ordinal (mesothermic > ectothermic).

*Feeding mechanism*: Elasmobranchs can be roughly divided into two groups with regard to their feeding strategies:

- Macropredators (i.e., macrophagous): We assigned a "macropredatory" feeding mechanism to all species that are known to actively hunt or catch prey. This trait designation includes bottom and suction feeders that prey upon invertebrates and/or fish.

- Filter feeders (i.e., microphagous or planktivorous). A “filter-feeding” mechanism was assigned to all species that consume plankton or krill via suspension feeding, bulk, or selective filter-feeding, which included three species of sharks (*Cetorhinus maximus, Megachasma pelagios* and *Rhincodon typus*) and rays from the family Mobulidae [18].

This trait was ordinal (macropredator > micropredator).

*Diet:* We classified the diet of each species based on the principal type of food it consumes as an adult. Diets were expressed as broad categories including:

- High vertebrates (e.g., marine mammals and seabirds)
- Fish
- Invertebrates
- Plankton.

It should be noted that many shark species are opportunistic generalists feeding on a variety of food[19], with the prey preference of some species varying seasonally [20, 21, 22]. Previous works attempting to standardise sharks’ diet composition have outlined up to 11 prey categories [23]. However, even this fine categorisation scheme fails to fully capture the complex dietary spectrum of sharks. For example, the bonnethead shark (*Sphyrna tiburo*) primarily feeds on crustaceans [24], but also consumes seagrass [25]. Similarly, the whale shark (*R. typus*) mostly filter-feeds on plankton, but it has also been reported to feed on macroalgae [26]. Although our broad prey preference categorisation scheme does not account for the full range of prey sharks can have during their lifetime, it allows capture of the most common diets of shark species. For the analyses, this trait was re-coded to be binary and fuzzy, as a single species could have multiple values (e.g., *Galeocerdo cuvier*, diet plankton = 0, diet inverts = 1, diet fish = 1, diet high verts = 1).

**MPA analyses.**

The following MPA designations were removed from our analyses:

- Wetland Protected Area
- Forest Nature Reserve
- Waterfowl gathering area
- Seabird Sanctuary
- Managed Flora Reserve
- Migratory Bird Sanctuary
- Shell Reserve
- Archaeological Preserve State Park
- Botanical State Park
- Bird Reserve
- Special Botanical Reserve
- Special Use Forest

<u>Definitions of IUCN categories for MPAs (from Day et al.)</u>[27]

*Ia Strict Nature Reserve:* Category Ia are strictly protected areas set aside to protect biodiversity and also possibly geological/geomorphological features, where human visitation, use and impacts are strictly controlled and limited to ensure protection of the conservation values. Such protected areas can serve as indispensable reference areas for scientific research and monitoring.

*Ib Wilderness Area:* Category Ib protected areas are usually large unmodified or slightly modified areas, retaining their natural character and influence without permanent or significant human habitation, which are protected and managed so as to preserve their natural condition.

*II National Park:* Category II protected areas are large natural or near natural areas set aside to protect large-scale ecological processes, along with the complement of species and ecosystems characteristic of the area, which also provide a foundation for environmentally and culturally compatible, spiritual, scientific, educational, recreational, and visitor opportunities.

*III Natural Monument or Feature:* Category III protected areas are set aside to protect a specific natural monument, which can be a landform, sea mount, submarine cavern, geological feature such as a cave or even a living feature such as an ancient grove. They are generally quite small protected areas and often have high visitor value.

*IV Habitat/Species Management Area:* Category IV protected areas aim to protect particular species or habitats and management reflects this priority. Many Category IV protected areas will need regular, active interventions to address the requirements of particular species or to maintain habitats, but this is not a requirement of the category.

*V Protected Landscape/ Seascape:* A protected area where the interaction of people and nature over time has produced an area of distinct character with significant, ecological, biological, cultural and scenic value: and where safeguarding the integrity of this interaction is vital to protecting and sustaining the area and its associated nature conservation and other values.

*VI Protected area with sustainable use of natural resources:* Category VI protected areas conserve ecosystems and habitats together with associated cultural values and traditional natural resource management systems. They are generally large, with most of the area in a natural condition, where a proportion is under sustainable natural resource management and where low-level non-industrial use of natural resources compatible with nature conservation is seen as one of the main aims of the area.

## Supplementary Figures

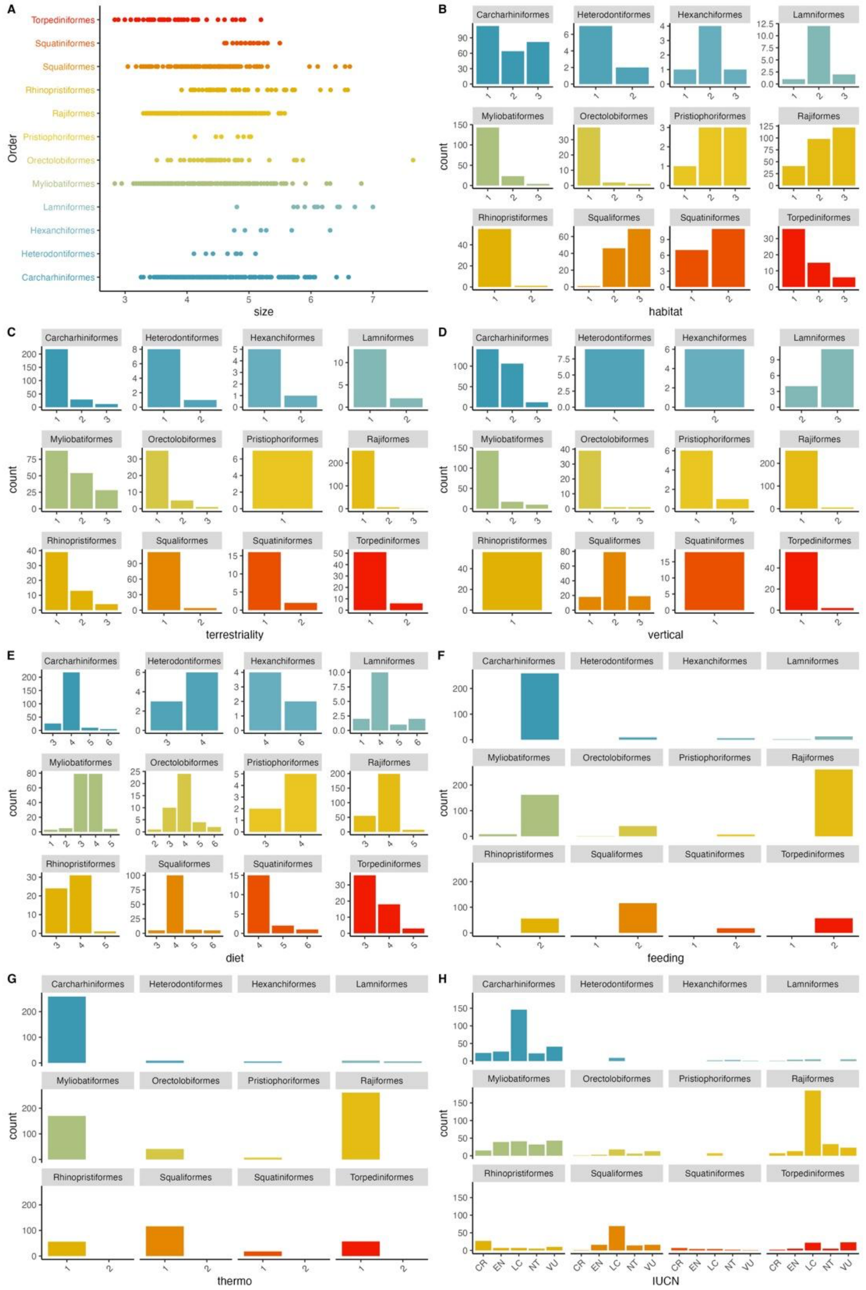


**Figure S1. Distribution of trait categories per Order. A.** Maximum body size in cm (log-transformed). **B.** Habitat: 1 = coastal; 2 = coastal and oceanic; 3 = oceanic. **C.** Terrestriality: 1 = No (fully marine); 2 = brackish; 3 = Freshwater. **D.** Vertical position: 1 = benthic; 2 = benthopelagic; 3 = pelagic. **E.** Diet: 1 = plankton; 2 plankton and fish; 3 = invertebrates; 4 = invertebrates and fish; 5 = fish; 6 = inverts, fish and high invertebrates. **F.** Feeding mechanism: 1 = filter feeders; 2 = macropredators. **G.** Thermoregulation: 1 = ectothermic; 2 = mesothermic. **H.** IUCN Red list threat status. All trait values are shown after multiple imputations had been performed, and converted to factors to facilitate visualisation, except for size (A) (see main text and Dataset S2).

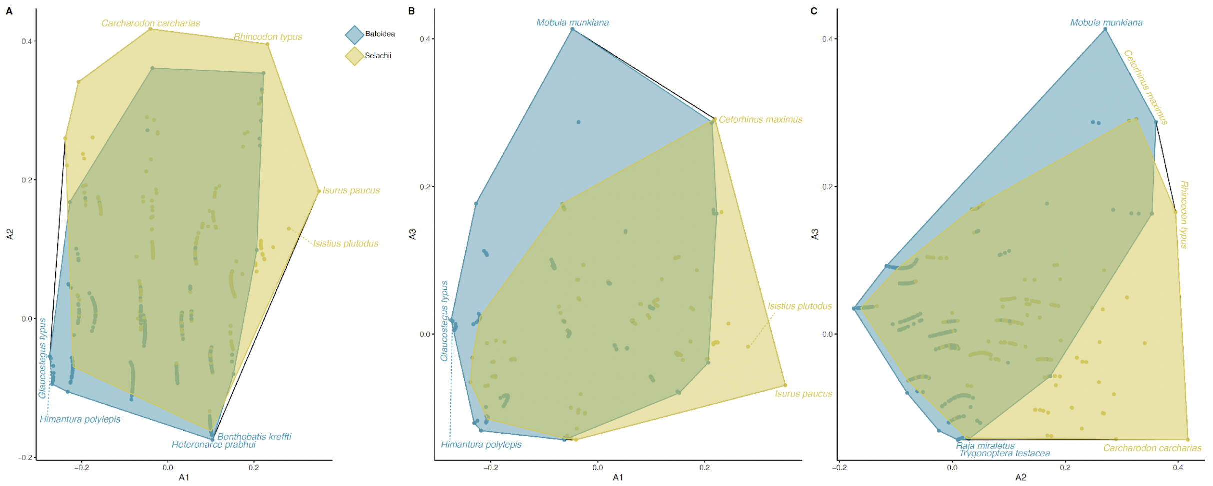


**Figure S2. Functional space occupied by the two elasmobranch superorders.** Blue space denotes Batoids (rays and skates), yellow space denotes Selachii (sharks). **A.** PCoA 1 and PCoA 2. **B.** PCoA 1 and PCoA 3. **C.** PCoA 2 and PCoA 3. Labelled species are those with the most extreme values along each axis.

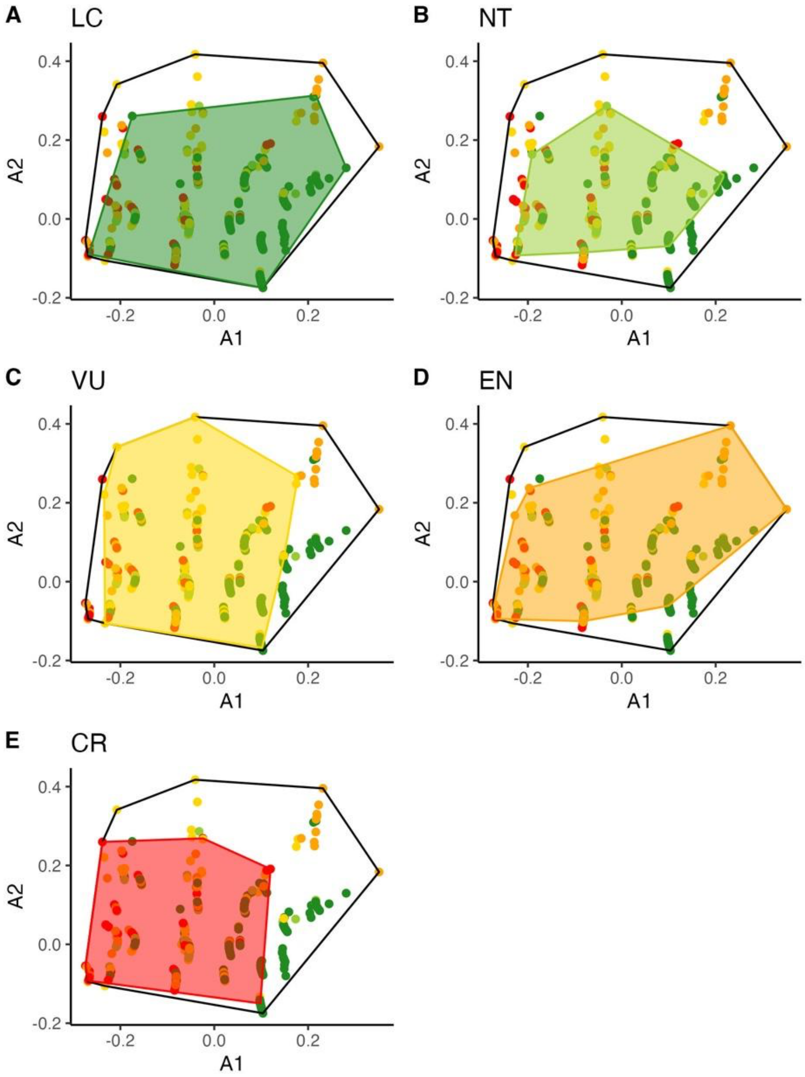


**Figure S3. Functional space occupied by the different IUCN Red List categories.** Spaces show 2 first dimensions (A1 and A2). **A.** LC = Least Concern. **B.** NT = Near Threatened. **C.** VU = Vulnerable. **D.** EN = Endangered. **E.** CR = Critically Endangered.

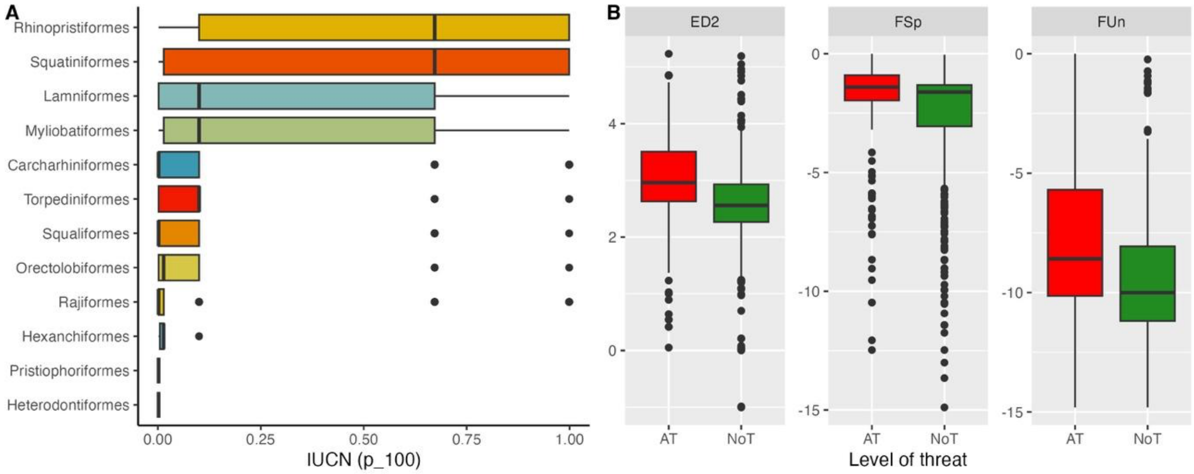


**Figure S4. Elasmobranch extinction risk. A.** Extinction probabilities in 100 years ("P100") as provided by IUCN (see main text) per order. **B.** Distribution of values for evolutionary distinctiveness (ED); functional specialisation (FSp); and functional uniqueness (FUn) across level of threat (AT = All threatened species [IUCN = VU, EN and CR]; NoT = Non-threatened species [LC and NT]). Wilxon-test $p < 0.05$.

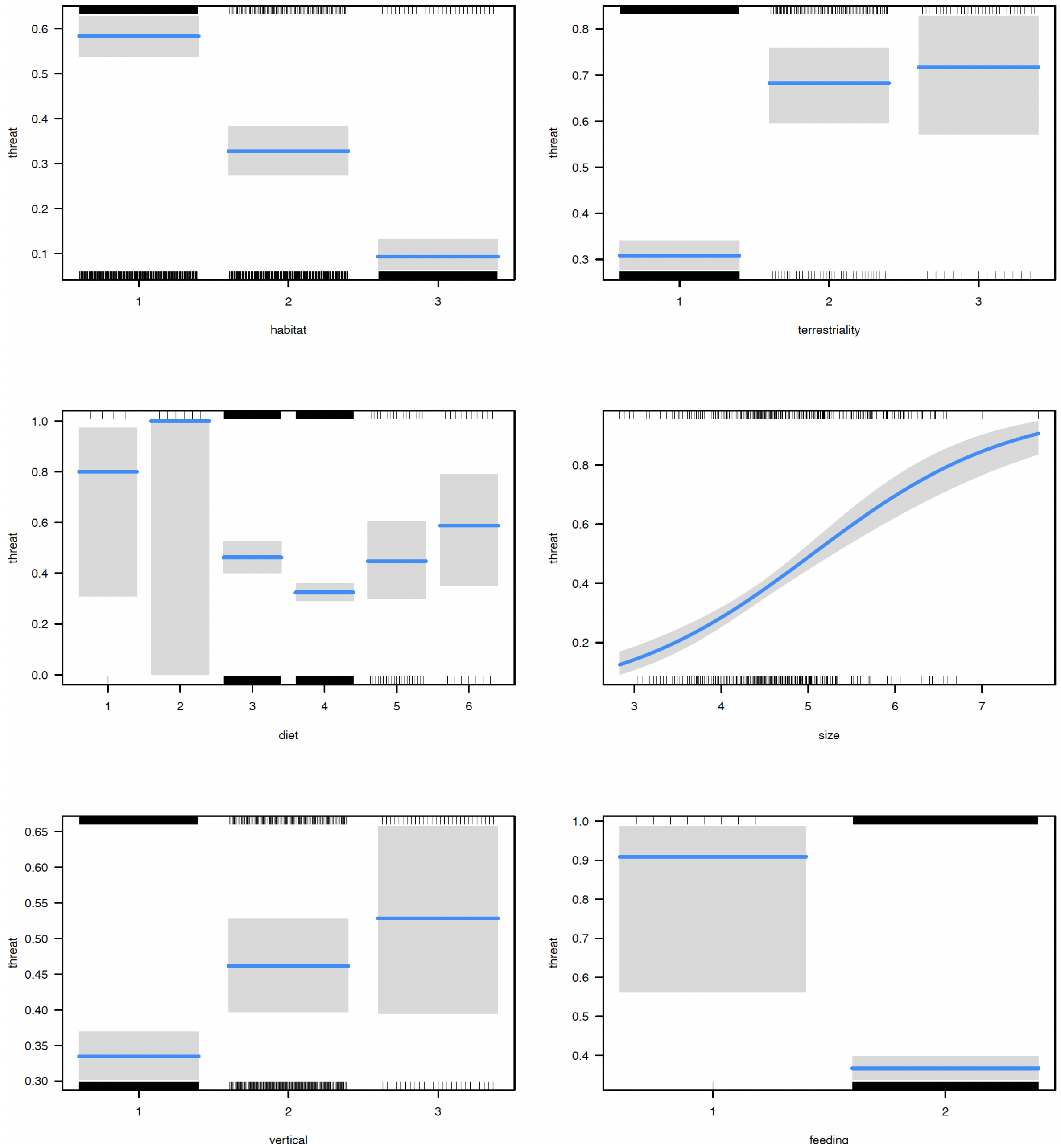


**Figure S5. Trait correlates of extinction risk (as provided by Binomial Generalized Linear Models).** Vertical lines denote the individual threatened species (top) and not threatened (bottom). Y-axis represents the level of threat as provided by IUCN Red List status (see main text). Inference is drawn by dropping the interaction term ('drop1') from the full model (see Fig. S5). Trait values are as in Fig S1: *Habitat*: 1 = coastal; 2 = coastal and oceanic; 3 = oceanic. *Terrestriality*: 1 = No (fully marine); 2 = brackish; 3 = Freshwater. *Vertical position*: 1 = benthic; 2 = benthopelagic; 3 = pelagic. *Diet*: 1 = plankton; 2 plankton and fish; 3 = invertebrates; 4 = invertebrates and fish; 5 = fish; 6 = inverts, fish and high invertebrates. *Feeding mechanism*: 1 = filter feeders; 2 = macropredators. *Thermoregulation*: 1 = ectothermic; 2 = mesothermic

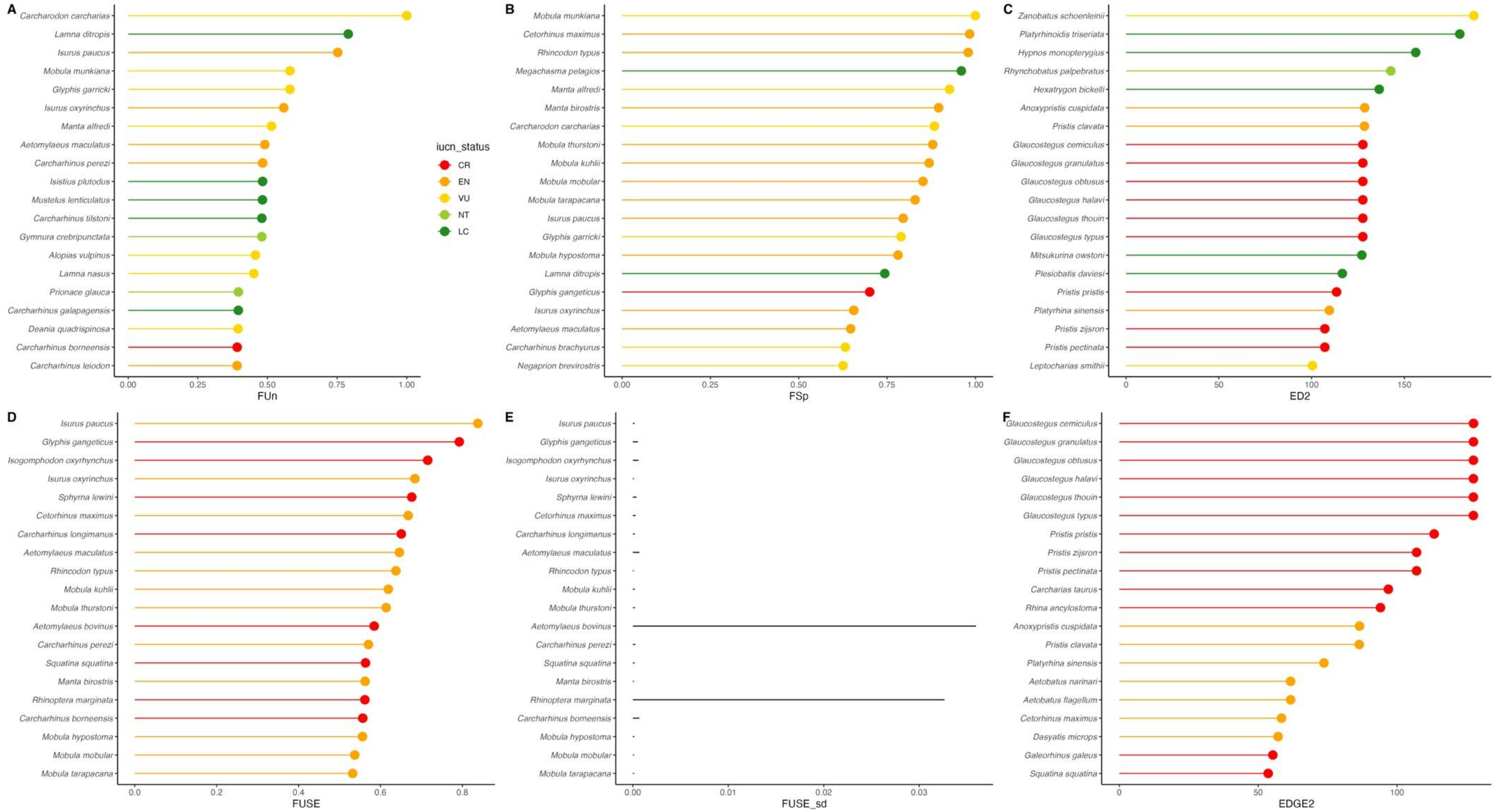


**Figure S6. Top 20 species**. **A.** Most functionally unique (FUn) species. **B.** Most functionally specialised (FSp). **C.** Most evolutionary distinct (ED). **D.** Highest-ranking FUSE species. **E.** Standard deviation of FUSE values when computing this metric using all imputed trait datasets (see Methods). **F.** Highest-ranking EDGE species.

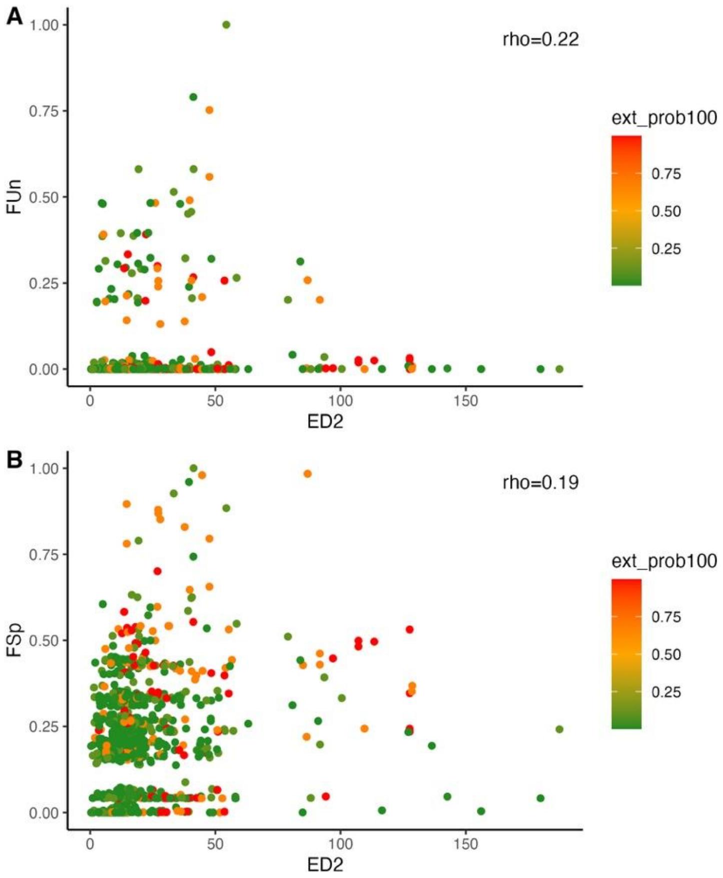


**Figure S7. Spearman correlation between species' functional and evolutionary distinctiveness. A.** Correlation between species' functional uniqueness (FUn) and evolutionary distinctiveness (ED). **B.** Between species' functional specialisation (FSp) and ED2. Colours represent IUCN Red List status, converted to extinction probabilities in 100 years (see main text).

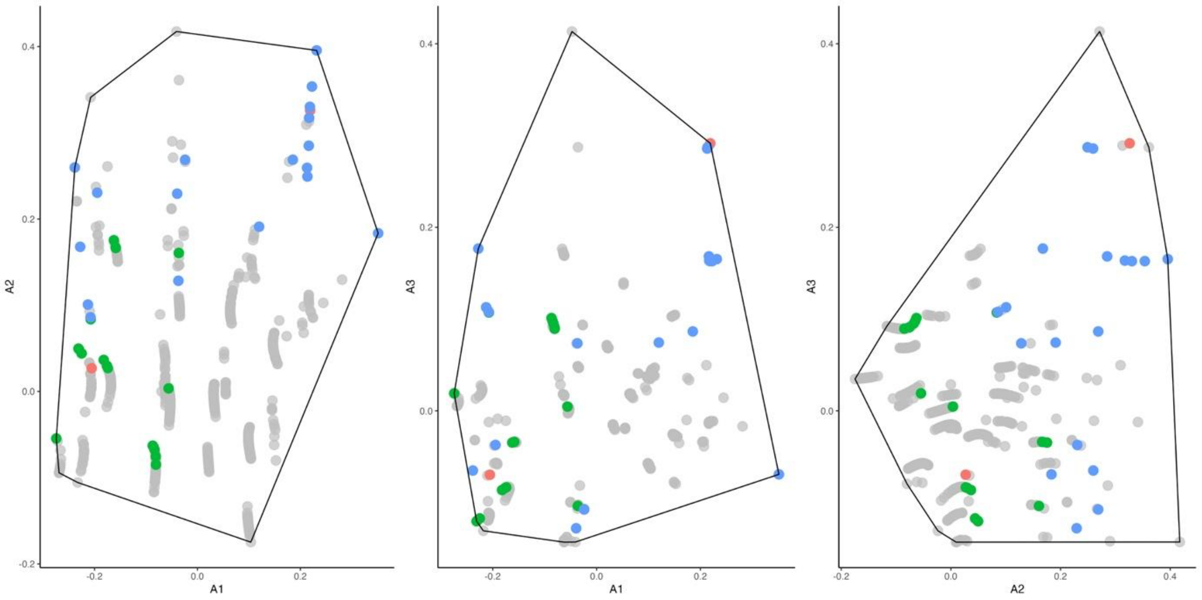


**Figure S8. Three-dimensional elasmobranch functional space.** Text denotes top five FUSE (blue) and EDGE (green) species in the global elasmobranch functional space. Pink colour denotes shared species between top 20 FUSE and EDGE.

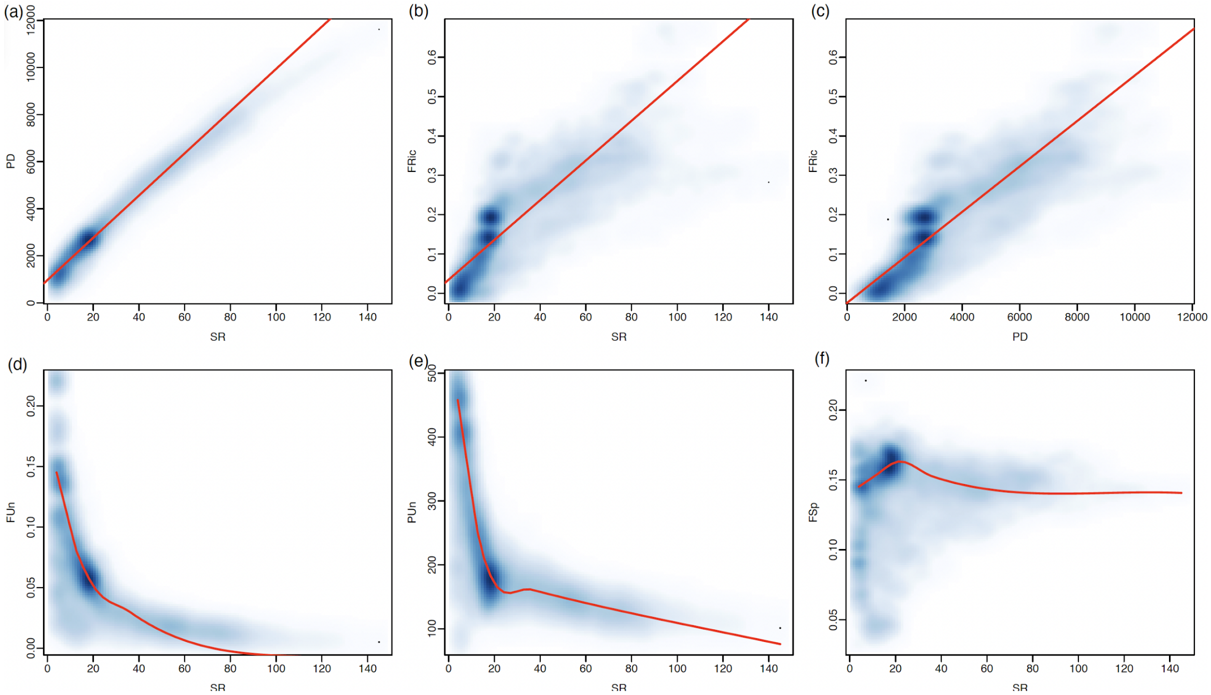


**Figure S9. Relationships between biodiversity indices. A.** Species richness (SR) *vs.* Phylogenetic Diversity (PD). **B.** SR *vs.* Functional Richness (FRic). **C.** SR *vs.* PD. D. SR *vs.* FUn. **E.** SR *vs.* Phylogenetic Uniqueness (PUn). **F.** SR *vs.* Functional Specialisation (FSp). The red line corresponds to the fit of a linear regression model between the indices considered in A-C and of a loess regression spline for D, E and F. The blue gradient shows the density of points (the darker the blue the more points overlap each other).

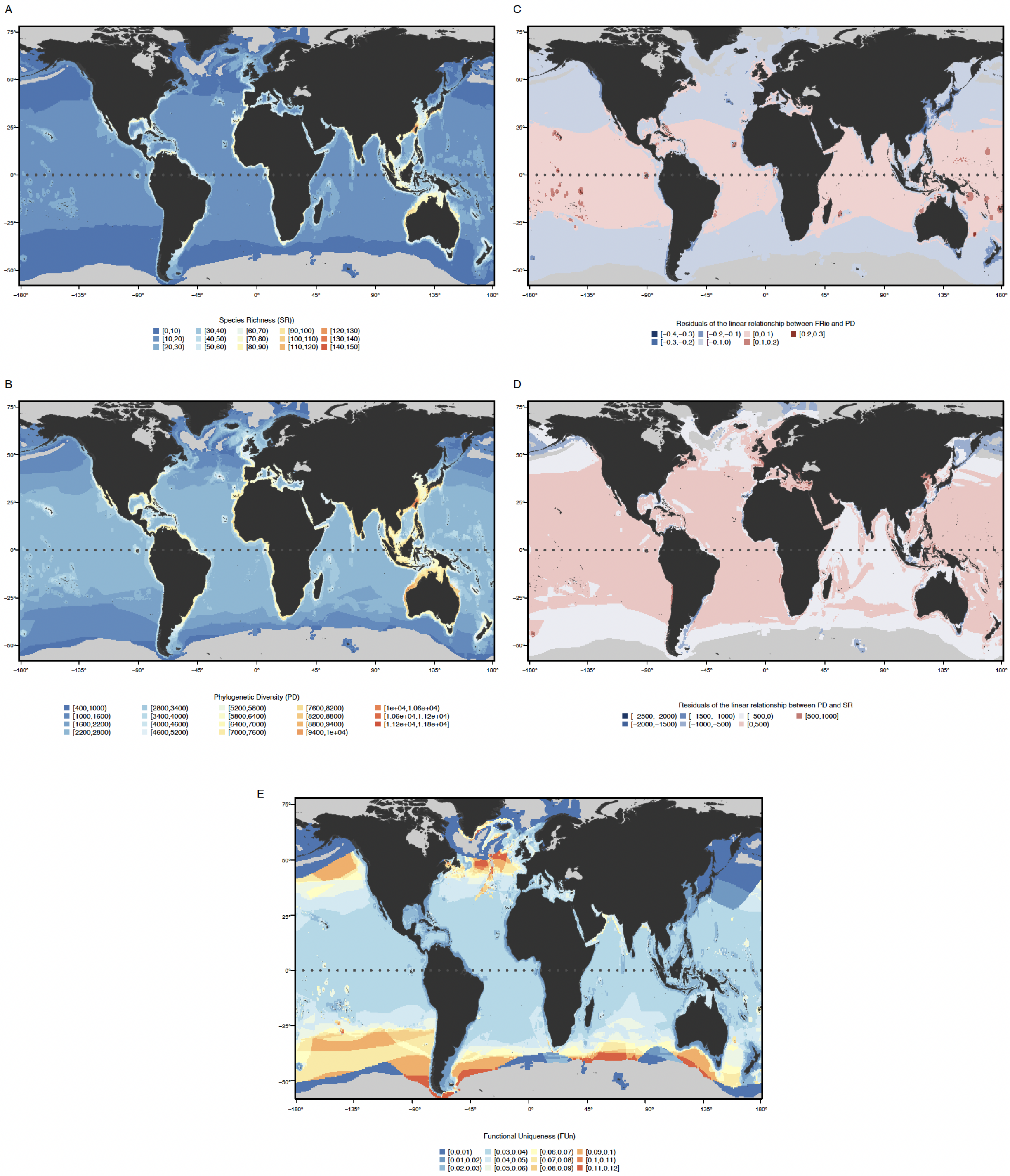


**Figure S10. Other facets of elasmobranch biodiversity and residuals of the relationship between metrics. A.** Species richness (SR) per cell. **B.** Mean (based on 100 phylogenetic trees, see main text) phylogenetic diversity (PD) values per cell. **C.** Residuals of the relationship between PD and Functional Richness (FRic) derived from a linear regression model (Fig. S9). **D**. Residuals of the relationship between PD and SR derived from a linear regression model (Fig. S9). **E.** Functional uniqueness (FUn) calculated as the mean functional distance between each species in a grid cell and its nearest neighbour within the global pool of species. All maps have been created using the R environment.

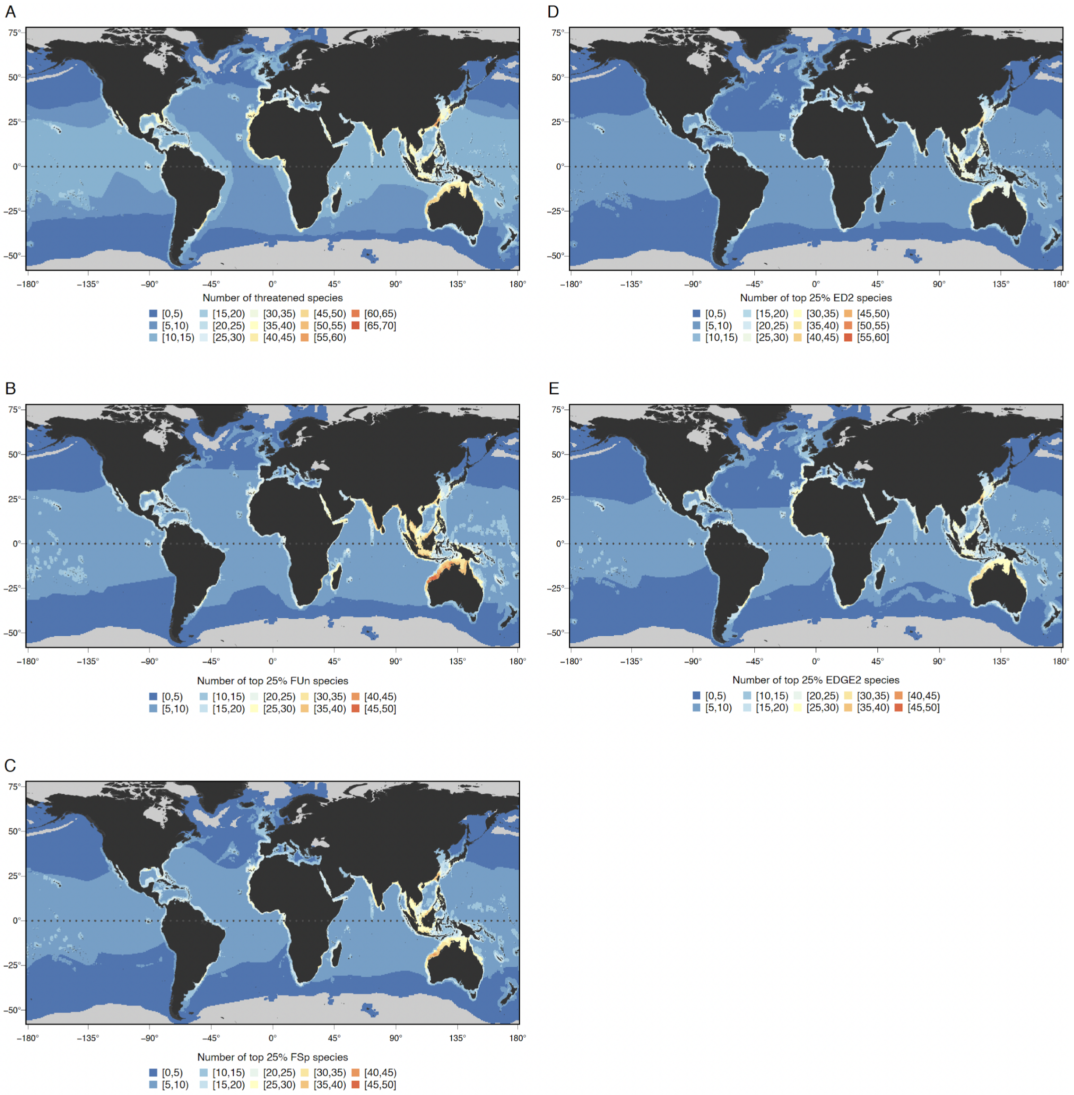


**Figure S11. Distribution of species-based metrics. A.** Number of threatened species (IUCN: CR, EN, VU) per cell. **B.** Number of top 25% functionally unique (FUn) species. **C.** Number of top 25% functionally specialised (FSp) species. **D.** Number of top 25% evolutionary distinct (ED2) species. **E** Number of top 25% EDGE2 species. All maps have been created using the R environment.

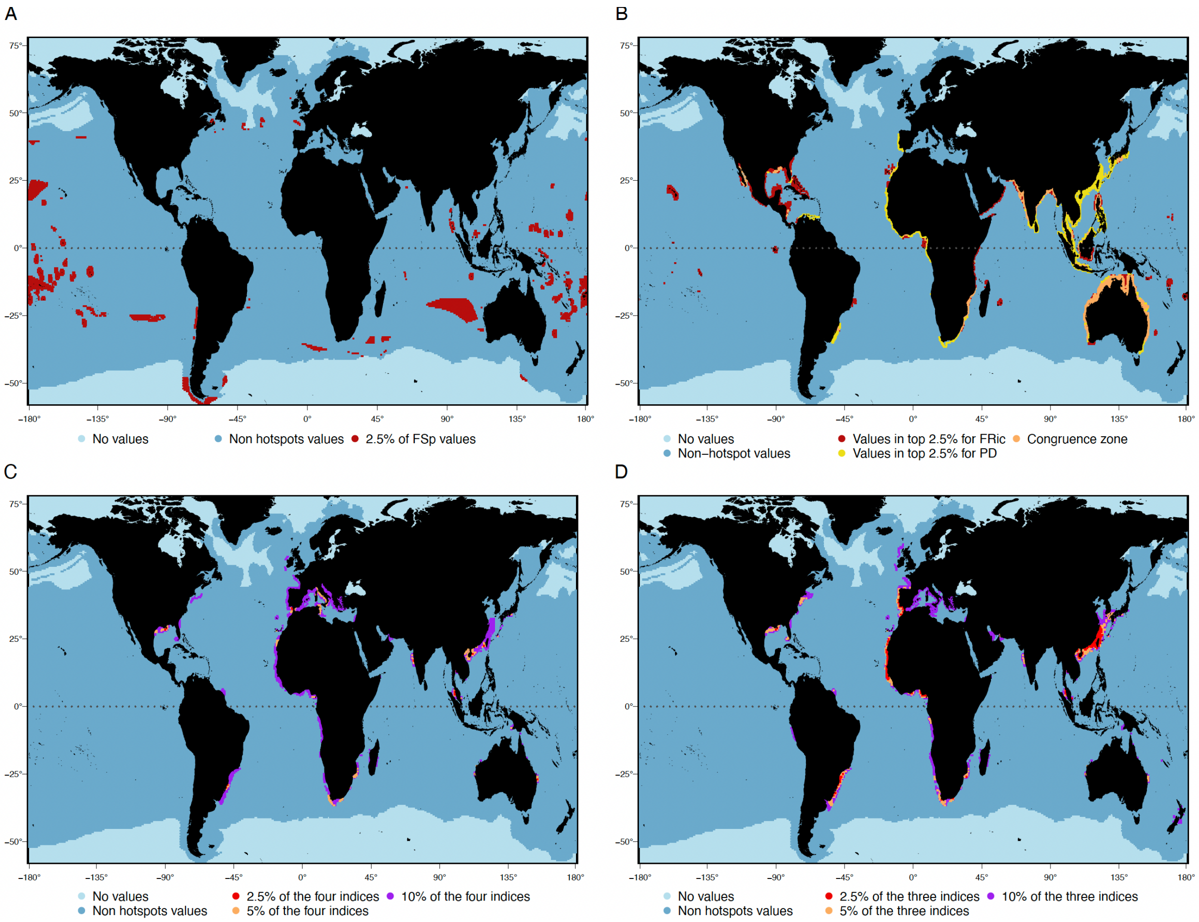


**Figure S12. Hotspots of elasmobranch diversity and their overlap. A.** Hotspots (top 2.5%) of Functional Specialisation (FSp). **B.** Overlap between hotspots (top 2.5% values) of Functional Richness (FRic) and Phylogenetic Diversity (PD). **C.** Congruence between Species Richness (SR), PD, FRic and fishing impact using 2.5%, 5% and 10% hotspot thresholds (see main text). **D.** Congruence between FUSE, EDGE2 and fishing impact using 2.5%, 5% and 10% hotspot thresholds (see main text). All maps have been created using the R environment.

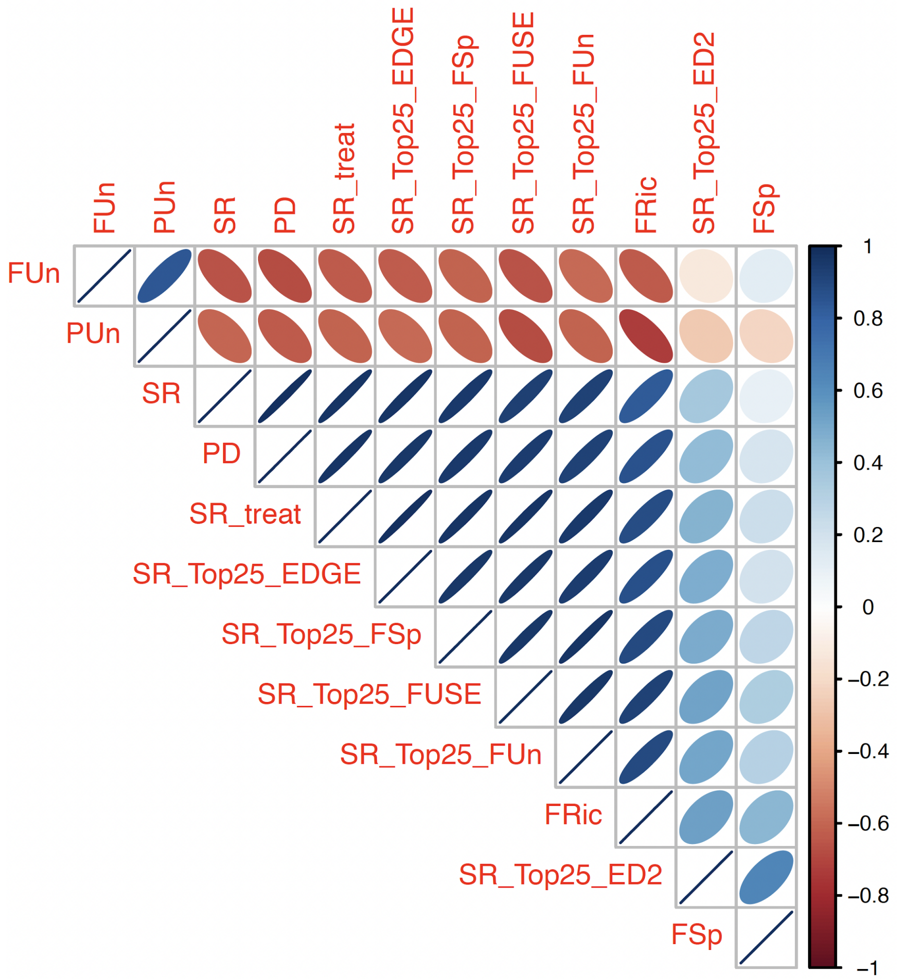


**Figure S13. Draftsman plot for all the biodiversity metrics.** Colour gradient shows the Pearson's correlation coefficient from negative (red) to positive (blue) associations. The width of the disk for each relationship represents the scatter around the correlation line for each relationship.

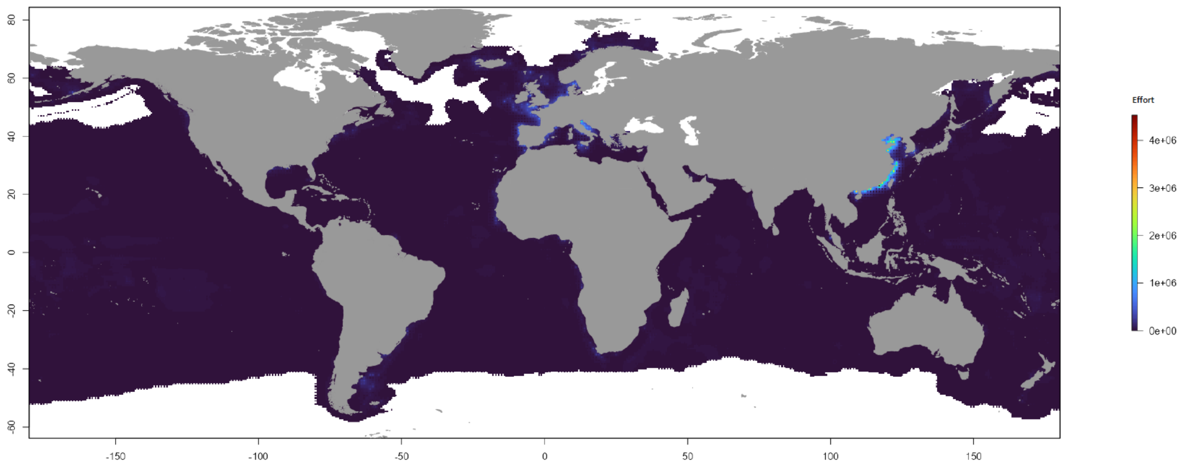


**Figure S14. Cumulative Fishing Pressures (2012-2020).** Data downloaded from Global Fishing Watch (https://globalfishingwatch.org; see main text). Map created using the R environment.

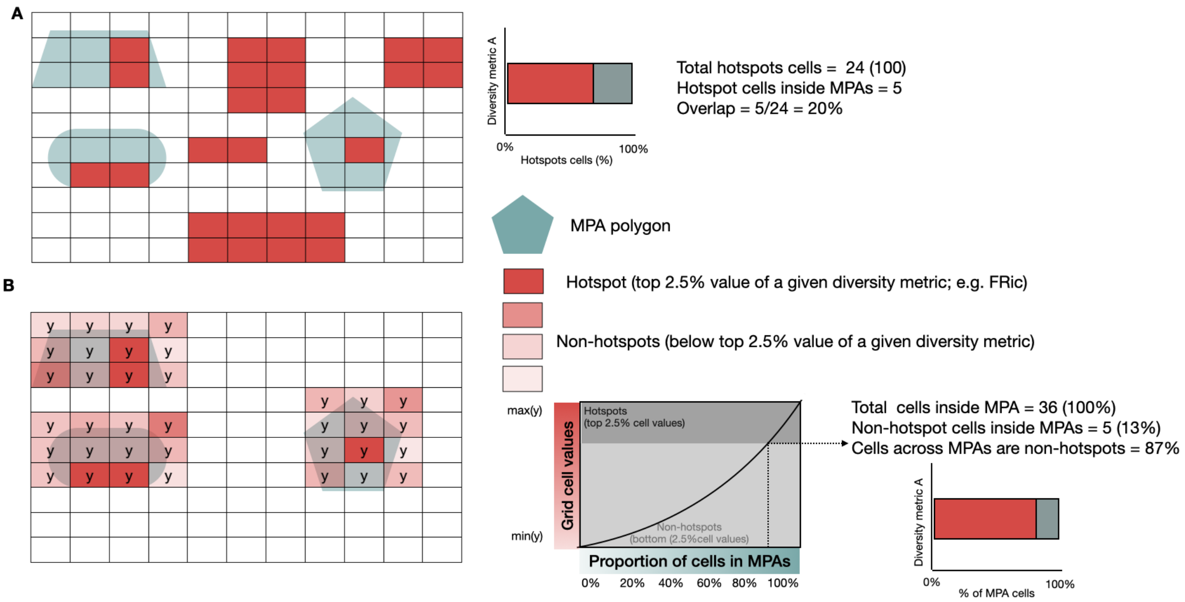


**Figure S15. Schematic representation of our assessment of the level of protection of elasmobranch biodiversity facets by MPAs. A.** Assessment of the overlap between hotspots and MPAs. **B.** Assessment of the coverage of hotspots by MPAs (see main text).

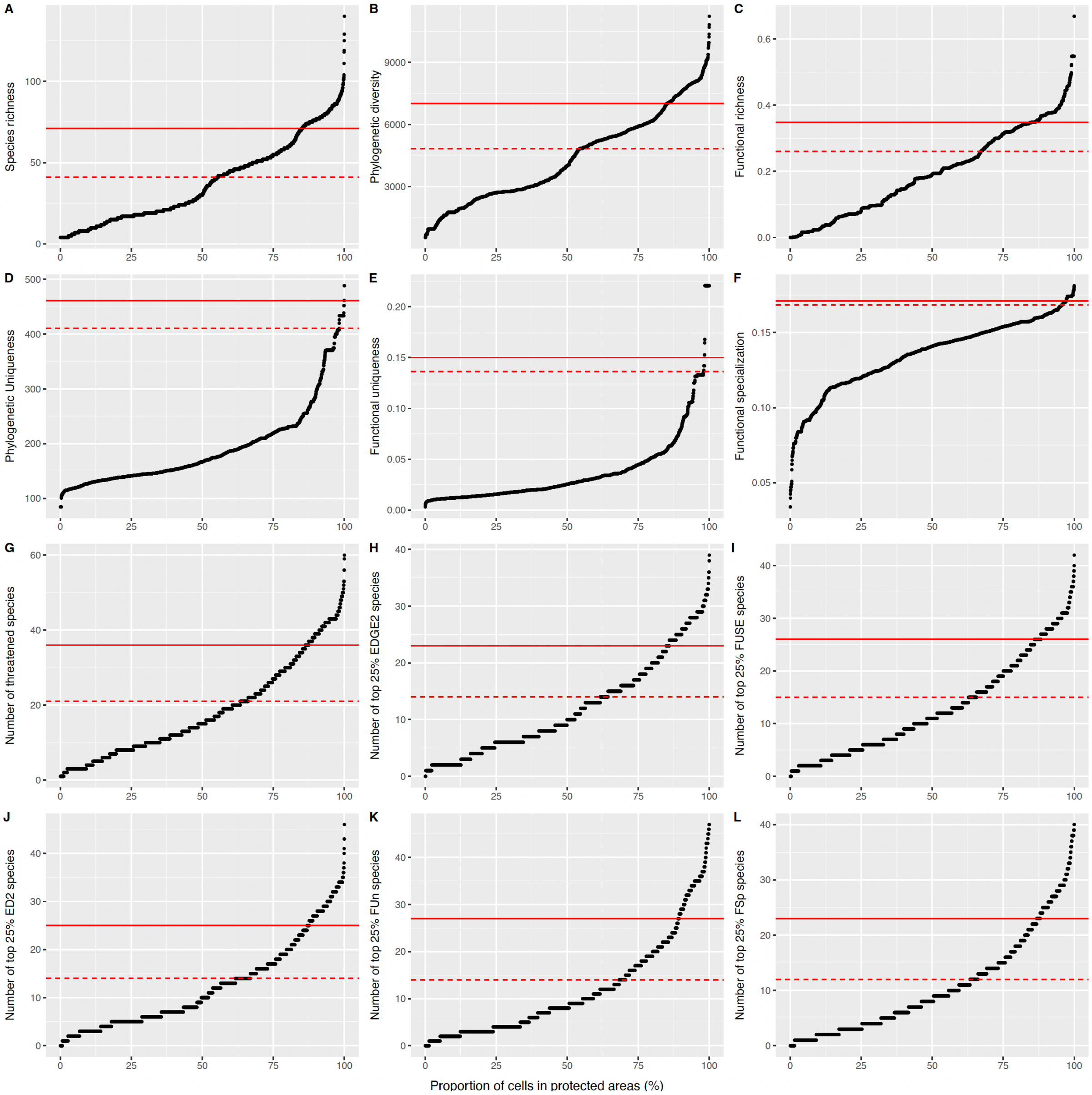


**Figure S16. Elasmobranch biodiversity represented in the world's MPAs.** Each plot (**A-L**) gives the values of the corresponding diversity index represented within protected grid cells of the Exclusive Economic Zone. Y-axes show the values of each index within each protected grid cells ranked from lowest to highest, whereas the x-axis shows the cumulative percentage of protected cells in the world's Exclusive Economic Zones. The full and dashed lines indicate the top 2.5% and 10% hotspot values of each index, respectively. As such, all cells above the red lines are considered hotspots (see Fig. S17 for simplified version of this figure).

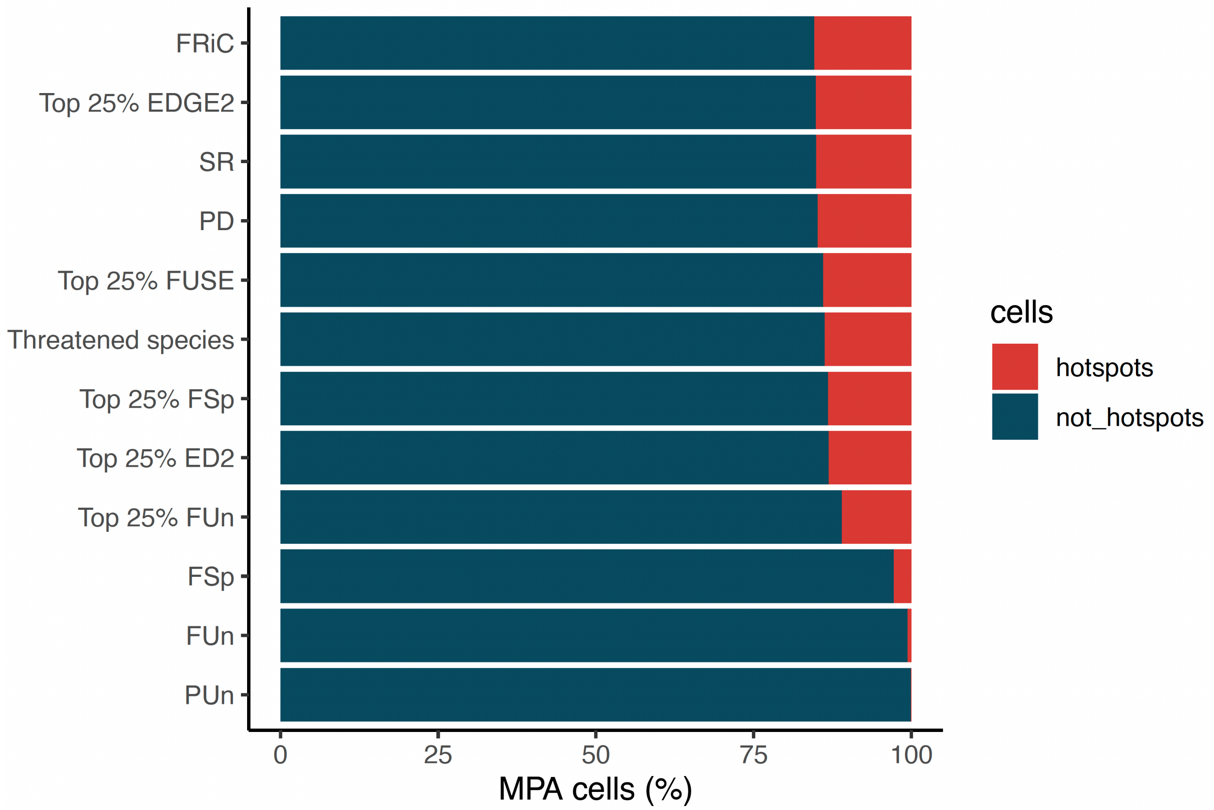


**Figure S17. Elasmobranch biodiversity coverage by the world's MPAs.** From Fig. S16, here we show the intersect between solid line (2.5% top values = hotspots as defined here) and the amount of these cells across all MPAs that are (red bars) or not (blue bars) hotpots of elasmobranch diversity (also see main text).

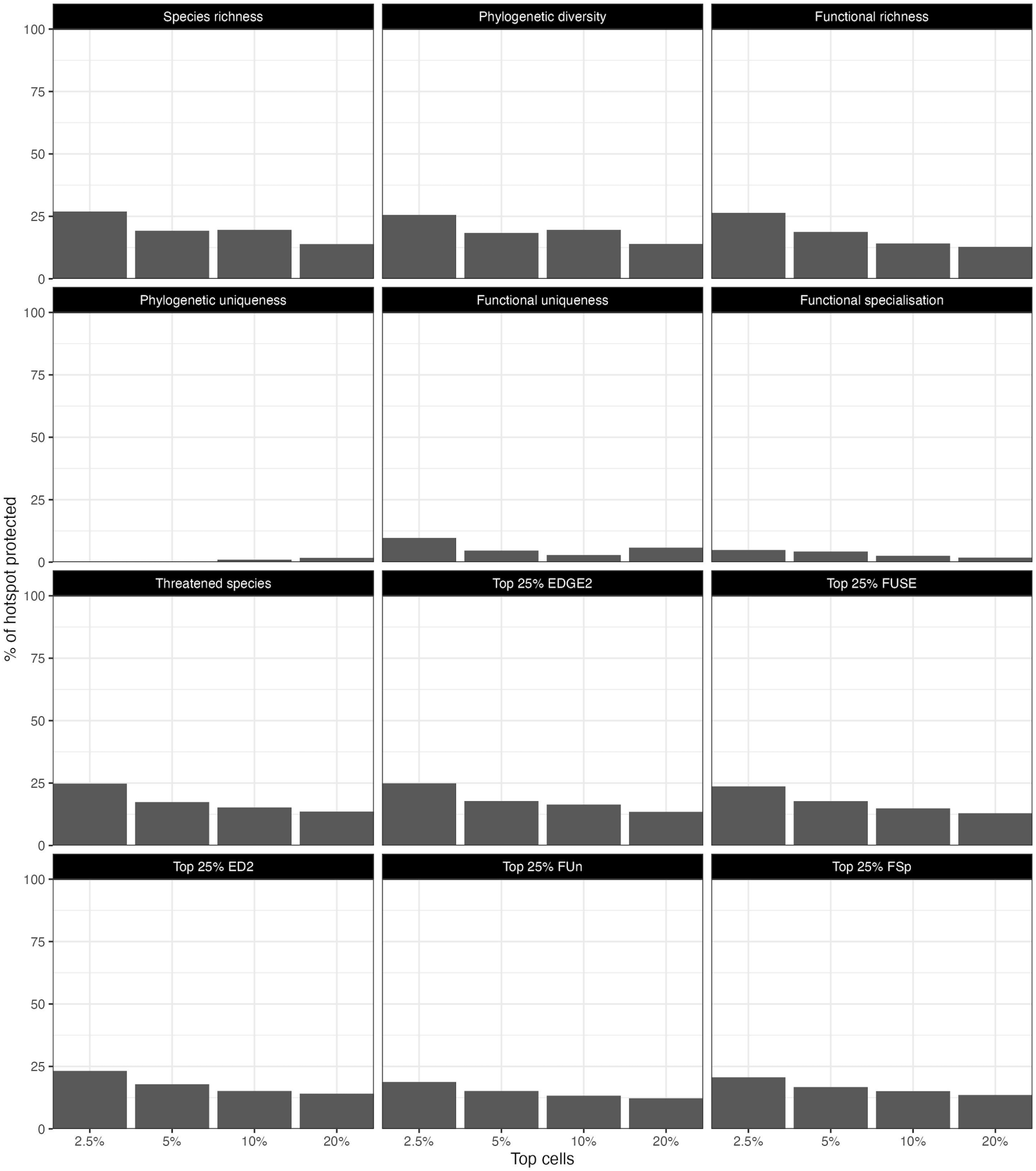


**Figure S18. Percentage of biodiversity hotspots protected by Marine Protected Areas (MPAs).** Hotspots are defined as cells with top 2.5%, 5%, 10% and 20% values for each corresponding diversity index. Different facets of biodiversity are considered, including species richness (SR); phylogenetic diversity (PD); functional richness (FRic); evolutionary distinctiveness (ED), functional uniqueness (FUn); and functional specialisation (FSp).

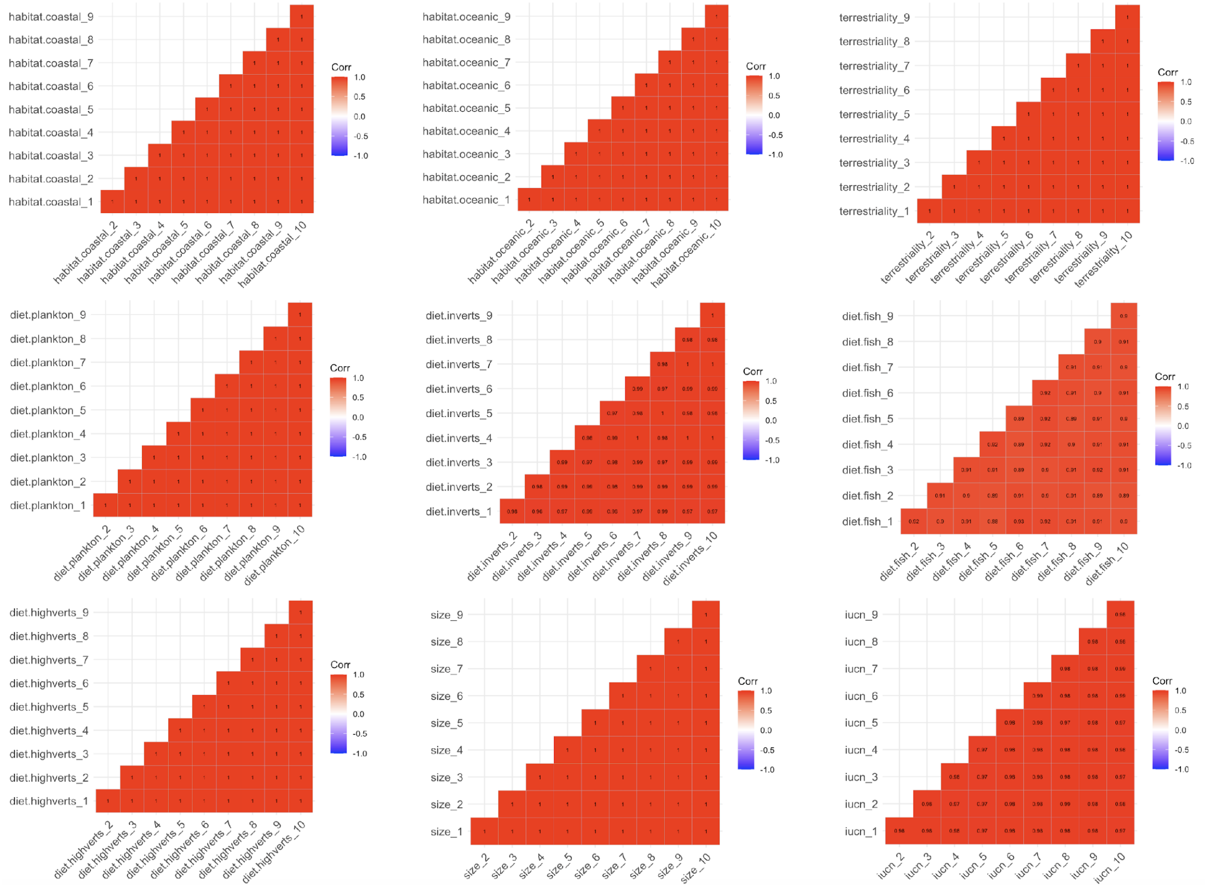

**Figure S19. Correlation between imputed trait values across 10 iterations.** Numbers in the axes denote iterations from 1 to 10. Numbers inside the tiles denote the correlation coefficient.

**Supplementary Tables**

**Table S1. Quality of the functional spaces built according to an increasing number of axes. Values correspond to the mean squared deviation (mSD) given by "quality_space" function[28]). The total explained inertia is also given for each functional space.** Values in bold denotes the number of dimensions used in the analyses.

| | mSD | Total inertia (%) |
|---|---|---|
| 2 axes | 0.001783 | 62.3% |
| 3 axes | **0.001527** | **75.0%** |
| 4 axes | 0.001532 | 84.2% |
| 5 axes | 0.00163 | 90.6% |
| 6 axes | 0.001827 | 93.6% |
| 7 axes | 0.001843 | 95.9% |
| 8 axes | 0.001957 | 97.8% |
| 9 axes | 0.00142 | 99.1% |
| 10 axes | 0.001468 | 99.9% |

**Table S2. Relationships between each PCoA axis and the original trait values.** The table shows the coefficient of determination (r-squared ranging from 0 to 1) derived from robust regression models relating species' coordinates on each PCoA axis and the original trait values (see methods). Bold values indicate the trait explaining the highest variance in each axis.

| Trait | Axis 1 | Axis 2 | Axis 3 |
|---|---|---|---|
| Habitat | **0.95411625** | 0.13716557 | 0.26964338 |
| Terrestrially | 0.49603255 | 0.0663043 | 0.18695039 |
| Diet | 0.07807242 | 0.56649386 | **0.56716945** |
| log(size) | 0.0155617 | 0.40499084 | 0.02224068 |
| Vertical position | 0.08999376 | **0.65227249** | 0.02716339 |
| Thermoregulation | 0.01478439 | 0.08320989 | 0.00064146 |
| Feeding | 0.0282324 | 0.18443451 | 0.14323688 |

**Table S3. Functional diversity and species richness by elasmobranch Superorders and Orders.** SR denotes species richness. FRic denotes functional richness (% of space occupied relative to full functional space). FSp denotes functional specialisation (mean distance of species to the centroid of functional space for each clade). FUn denotes functional uniqueness (species' mean distance to closest five neighbours of the same order). Both FUn and FSp are therefore calculated based on the functional space of each clade, unlike in Table S4 (see below). Bold denotes highest values and grey color denotes lowest values.

| Superorder/ Order | SR | FRic (%) | FSp | FUn |
|---|---|---|---|---|
| Batoidea | **544** | 59.9 | 0.12924965 | 0.00341275 |
| Selachii | 471 | 68.2 | 0.1343549 | 0.00756177 |
| Carcharhiniformes | 259 | 27.9 | 0.13467287 | 0.00852115 |
| Heterodontiformes | 9 | 0.2 | **0.06354264** | 0.05350469 |
| Hexanchiformes | **6** | 0.1 | 0.0857954 | 0.11144476 |
| Lamniformes | 15 | 17.5 | **0.15555003** | **0.11797315** |
| Myliobatiformes | 170 | **53.7** | 0.1489323 | 0.01069126 |
| Orectolobiformes | 41 | 9.9 | 0.07925277 | 0.02961946 |
| Pristiophoriformes | 7 | 1 | 0.08752445 | 0.10311523 |
| Rajiformes | **261** | 6.6 | 0.08447693 | **0.00264376** |
| Rhinopristiformes | 56 | 1.1 | 0.09801416 | 0.00554228 |
| Squaliformes | 116 | 7.1 | 0.0834602 | 0.01000536 |
| Squatiniformes | 18 | **0.1** | 0.06359377 | 0.02708985 |
| Torpediniformes | 57 | 7 | 0.1020273 | 0.01901152 |

**Table S4. Per-species functional specialization (FSp), uniqueness (FUn), FUSE, evolutionary distinctiveness (ED2) and EDGE2. Mean values across orders.** Bold denotes highest values and grey colour denotes lowest values. Unlike Table S3, FUn and FSp were computed per species, and then we calculated the mean of each Order. Bold denotes highest values and gray color denotes lowest values.

| Order | SR | FSp | FUn | FUSE | ED2 | EDGE2 |
|---|---|---|---|---|---|---|
| Carcharhiniformes | 259 | 0.21734301 | 0.030211 | 0.05340922 | 18.8221665 | 3.90502195 |
| Heterodontiformes | 9 | 0.12134032 | **0.00107448** | **0.00021405** | 21.9249244 | **0.03834327** |
| Hexanchiformes | **6** | 0.26901884 | 0.06583971 | 0.0137095 | 36.8217499 | 1.52392881 |
| Lamniformes | 15 | **0.5712082** | **0.30894879** | **0.21790806** | 54.7186072 | 18.1291353 |
| Myliobatiformes | 170 | 0.32339592 | 0.02841851 | 0.11041511 | 24.5691062 | 6.74856251 |
| Orectolobiformes | 41 | 0.16816511 | 0.01993377 | 0.03776235 | 21.1179035 | 3.25565753 |
| Pristiophoriformes | 7 | 0.13649992 | 0.00113725 | 0.00024066 | 31.339568 | 0.05480802 |
| Rajiformes | **261** | 0.14511385 | 0.00460965 | 0.00603564 | **11.7166107** | 1.14951608 |
| Rhinopristiformes | 56 | 0.21926369 | 0.0031931 | 0.1315978 | **59.4226164** | **37.6132183** |
| Squaliformes | 116 | 0.25814998 | 0.02723231 | 0.02312726 | 18.4376336 | 2.89865441 |
| Squatiniformes | 18 | **0.05720518** | 0.01497418 | 0.05123859 | 27.5176361 | 18.6002909 |
| Torpediniformes | 57 | 0.21098766 | 0.00120045 | 0.02235589 | 21.5259882 | 3.48254785 |

**Table S5. Elasmobranch functional and species richness by IUCN status.** Bold denotes highest values.

| IUCN status | SR | FRic (%) |
|---|---|---|
| LC | **515** | 44.8 |
| NT | 122 | 20.5 |
| VU | 176 | **59.2** |
| EN | 118 | 54.7 |
| CR | 84 | 25.9 |
| Threatened (VU, EN and CR) | 378 | **97.6** |
| Not threatened (LC and NT) | **637** | 48.2 |

**Table S6. Binomial GLM output for the effect of traits on extinction probabilities.** LRT denotes likelihood ratio test and DF degrees of freedom. Bold values denote statistical significance.

| Trait | DF | LRT | Pr(>Chi) |
|---|---|---|---|
| habitat | 2 | 202.170348 | **1.26E-44** |
| terrestriality | 2 | 86.3301571 | **1.79E-19** |
| diet | 5 | 35.0654945 | **1.46E-06** |
| size | 1 | 82.8170912 | **9.00E-20** |
| vertical | 2 | 17.2025558 | **0.00018387** |
| thermoregulation | 1 | 5.43497465 | **0.01973738** |
| feeding | 1 | 14.1335886 | **0.00017028** |

**References cited in this Supplementary Information file**


1. Stein RW*, et al.* Global priorities for conserving the evolutionary history of sharks, rays and chimaeras. *Nature Ecology & Evolution* **2**, 288-+ (2018).

2. Boettiger C, Lang DT, Wainwright PC. rfishbase: exploring, manipulating and visualizing FishBase data from R. *Journal of Fish Biology* **81**, 2030-2039 (2012).

3. Froese R, Pauly D. FishBase World Wide Web electronic publication, Version (01/2017). *URL Www Fishbase Org Accessed* **1**, (2017).

4. Carey FG, Teal JM. MAKO AND PROBEAGLE WARM-BODIED SHARKS. *Comparative Biochemistry and Physiology* **28**, 199-204 (1969).

5. Block BA, Carey FG. WARM BRAIN AND EYE TEMPERATURES IN SHARKS. *Journal of Comparative Physiology B-Biochemical Systemic and Environmental Physiology* **156**, 229-236 (1985).

6. Bernal D*, et al.* Comparative studies of high performance swimming in sharks II. Metabolic biochemistry of locomotor and myocardial muscle in endothermic and ectothermic sharks. *Journal of Experimental Biology* **206**, 2845-2857 (2003).

7. Bernal D, Sepulveda CA. Evidence for temperature elevation in the aerobic swimming musculature of the common thresher shark, Alopias vulpinus. *Copeia*, 146-151 (2005).

8. Bernal D, Donley JM, Shadwick RE, Syme DA. Mammal-like muscles power swimming in a cold-water shark. *Nature* **437**, 1349-1352 (2005).

9. Bernal D, Carlson JK, Goldman KJ, Lowe CG. Energetics, metabolism, and endothermy in sharks and rays. *Biology of sharks and their relatives 2nd edition, CRC Press, Boca Raton*, 211-237 (2012).

10. Grady JM, Enquist BJ, Dettweiler-Robinson E, Wright NA, Smith FA. DINOSAUR PHYSIOLOGY Evidence for mesothermy in dinosaurs. *Science* **344**, 1268-1272 (2014).

11. Lowe CG, Goldman KJ. Thermal and bioenergetics of elasmobranchs: bridging the gap. *Environmental Biology of Fishes* **60**, 251-266 (2001).

12. Alexander R. Evidence of a counter-current heat exchanger in the ray, Mobula tarapacana (Chondrichthyes: Elasmobranchii: Batoidea: Myliobatiformes). *Journal of Zoology* **237**, 377-384 (1995).

13. Alexander R. Evidence of brain-warming in the mobulid rays, Mobula tarapacana and Manta birostris (Chondrichthyes: Elasmobranchii: Batoidea: Myliobatiformes). *Zoological Journal of the Linnean Society* **118**, 151-164 (1996).

14. Thorrold SR*, et al.* Extreme diving behaviour in devil rays links surface waters and the deep ocean. *Nature Communications* **5**, (2014).

15. Thums M, Meekan M, Stevens J, Wilson S, Polovina J. Evidence for behavioural thermoregulation by the world's largest fish. *Journal of the Royal Society Interface* **10**, (2013).

16. Meekan M, Fuiman L, Davis R, Berger Y, Thums M. Swimming strategy and body plan of the world's largest fish: implications for foraging efficiency and thermoregulation. *Frontiers in Marine Science* **2**, 64 (2015).

17. Watanabe Y, Goldman K, Caselle J, Chapman D, Papastamatiou Y. Comparative analyses of animal-tracking data reveal ecological significance of endothermy in fishes. *Proceedings of the National Academy of Sciences of the United States of America* **112**, 6104-6109 (2015).

18. Paig-Tran E, Summers A. Comparison of the Structure and Composition of the Branchial Filters in Suspension Feeding Elasmobranchs. *Anatomical Record-Advances in Integrative Anatomy and Evolutionary Biology* **297**, 701-715 (2014).

19. Wetherbee BM, Cortés E. *Food consumption and feeding habits*. CRC Press (2004).

20. MacNeil MA, Skomal GB, Fisk AT. Stable isotopes from multiple tissues reveal diet switching in sharks. *Marine Ecology Progress Series* **302**, 199-206 (2005).

21. Dicken ML*, et al.* Diet and trophic ecology of the tiger shark (Galeocerdo cuvier) from South African waters. *PLoS One* **12**, e0177897 (2017).

22. Baremore IE, Murie DJ, Carlson JK. Seasonal and size-related differences in diet of the Atlantic angel shark Squatina dumeril in the northeastern Gulf of Mexico. *Aquatic Biology* **8**, 125-136 (2010).

23. Cortés E. Standardized diet compositions and trophic levels of sharks. *ICES Journal of Marine Science* **56**, 707-117 (1999).

24. Mara KR, Motta PJ, Huber DR. Bite force and performance in the durophagous bonnethead shark, Sphyrna tiburo. *Journal of Experimental Zoology Part A: Ecological Genetics and Physiology* **313**, 95-105 (2010).

25. Leigh SC, Papastamatiou YP, German DP. Seagrass digestion by a notorious 'carnivore'. *Proceedings of the Royal Society B* **285**, (2018).

26. Meekan M, Virtue P, Marcus L, Clements K, Nichols P, Revill A. The world's largest omnivore is a fish. *Ecology*, e3818 (2022).

27. Day J*, et al. Guidelines for applying the IUCN protected area management categories to marine protected areas*. IUCN (2012).

28. Maire E, Grenouillet G, Brosse S, Villeger S. How many dimensions are needed to accurately assess functional diversity? A pragmatic approach for assessing the quality of functional spaces. *Global Ecology and Biogeography* **24**, 728-740 (2015).
