# Supplementary material for: Functional diversity of sharks and rays is highly vulnerable and supported by unique species and locations worldwide
Source: Nat Commun. 2023 Nov 24;14:7691. doi: 10.1038/s41467-023-43212-3 (PMC10673927; doi:10.1038/s41467-023-43212-3)
Supplement: Supplementary file 3 — Description of Additional Supplementary Files [file 41467_2023_43212_MOESM3_ESM.pdf]

Description of Additional Supplementary Files

Supplementary Data 1. Elasmobranch species and their traits as originally gathered (see main text). Sheet 1: Dataset. Sheet 2: Citations. Sheet 3: Full list of references.

Supplementary Data 2. Trait imputations. Sheet 1: Data used to perform imputations (input). Sheet 2: Modal values of imputed data across iterations (output).
